# The Impact of Orthodontic Adhesives on the Subgingival Microbiota During Early Fixed Appliance Therapy: A Pilot Study

**DOI:** 10.3390/medicina61050858

**Published:** 2025-05-07

**Authors:** Krisztina Martha, Esztella-Éva Kis, Izabella Éva Mureșan, Andrei Constantin Ioanovici

**Affiliations:** 1 Faculty of Dental Medicine, George Emil Palade University of Medicine, Pharmacy, Science and Technology of Târgu Mures, 38 Gh. Marinescu Str., 540139 Târgu Mureș, Romania; esztella.kis@umfst.ro (E.-É.K.); izabella-eva.muresan@umfst.ro (I.É.M.); 2 Faculty of Medicine, George Emil Palade University of Medicine, Pharmacy, Science and Technology of Târgu Mures, 38 Gh. Marinescu Str., 540139 Târgu Mureș, Romania; andrei.ioanovici@umfst.ro

**Keywords:** orthodontic fixed appliance, orthodontic adhesives, periodontopathogenic bacteria, PCR

## Abstract

*Background and Objectives*: The most common method for treating malocclusions today is fixed orthodontic therapy, during which brackets and tubes are bonded to the surface of the teeth, which makes oral hygiene difficult to maintain. Increased plaque retention, gingival bleeding, and gingivitis can be diagnosed in the early phases of treatment. The periodontal response to plaque accumulation can be explained by quantitative and qualitative changes in the subgingival microbiota. The aim of our research was to investigate the changes in the subgingival microbiota that occurred within 6–8 weeks after bonding when two different orthodontic adhesives were used. *Materials and Methods*: Thirty patients were followed; molar tubes were bonded with a composite (C) in fifteen cases, and in the other fifteen cases, they were bonded with glass ionomer cementum (GIC). A microbiological sample was taken from the gingival sulcus of the maxillary first molars at the time of appliance placement (T1) and 6–8 weeks (T2) after bonding. Bacterial DNA detection was performed using the micro-IDent^®^plus11 (Hain Lifescience GmbH, Germany) PCR (polymerase chain reaction) method. The statistical analysis included McNemar’s test to analyze the paired binary data and Fisher’s Exact Test to compare the prevalence of each of the 11 bacteria at T1 and, ultimately, T2 between the two studied groups. The Bonferroni correction was also applied. *Results*: When analyzing GIC vs. C at T1 and T2, none of the studied pathogens showed significant differences. *Conclusions*: Given the lack of statistical significance, these trends do not confirm a definitive impact of the procedure on bacterial presence. The increased presence of periodontal pathogens might suggest that bonding does not reduce the bacterial loading of subgingival microbiota. Less protective effects of the GIC intervention against *Tannerella forsythia* and *Eubacterium nodatum* bacteria were detected.

## 1. Introduction

The most common method for treating malocclusions today is orthodontic therapy with fixed appliances, during which brackets and tubes are bonded directly to the surface of the teeth, which makes it difficult for an individual to maintain oral hygiene. As a result, frequent complications of the treatment are increased plaque retention, gingival bleeding, and the appearance of periodontal pockets, and in some cases, gingivitis can be diagnosed during the early phase of the treatment [1]. The periodontal response in orthodontic treatment can be explained as a mechanical and/or chemical response to the bracket placement. Plaque accumulation can be explained by quantitative and qualitative changes in the subgingival microbiome [2].

Periodontal behavior is strongly related to the bonding procedure and materials used. Gingival inflammation will disturb the homeostasis of the gingival microbiome, and as well as local manifestations, it can lead to the development of general diseases [3].

Subgingival plaque was first characterized by Socransky et al., who grouped the forty most associated species into complexes. The grouping was performed based on species’ association with periodontitis, and they were color-labeled into green, yellow, orange, and red complexes. Green and yellow complexes are called “early colonizers” and can adhere to and prepare the aggregation of the bacteria in the orange complex. The orange complex is considered to comprise “bridge species”, which will help the connection between green and red bacteria; they are able to produce toxins and enzymes, all of which will increase the loss of periodontal attachment and pocket depth and create the perfect environment for colonization by bacteria in the red complex bacteria, the “late colonizers”. Red-complex bacteria are strongly associated with irreversible periodontal bleeding [4].

Molecular DNA-based techniques became standard for the identification of bacteria directly from gingival samples. The real-time PCR method and DNA-strip technology (DNA–DNA hybridization) are commercially available assays in microbiological laboratory procedures. For periodontopathogen identification and subsequently for oral microbiome study, the micro-IDent plus 11 test kit is a viable option. Marker pathogen analyses with micro-IDent^®^plus11 supply data on the quality and quantity of 11 periodontopathogenic species. By analyzing the subgingival sample, one of its greatest benefits is the risk assessment for a negative periodontal response [3].

The aim of our research was to examine the changes in the subgingival microbiome occurring 6–8 weeks after bonding, depending on the bonding material that was used.

## 2. Materials and Methods

Thirty-one patients were enrolled in this study; the subjects’ average age was 15.05 ± 2.16 years. Before sampling, the parents or legal representatives of the patients were informed about the research, and written consent was obtained from them. No additional fee was charged for periodontal pathogen bacteria analysis. The protocol was approved by the Research Ethics Committee of George Emil Palade University of Medicine, Pharmacy, Science, and Technology of Targu Mures in its resolution no. 3011/01.04.2024.

The research was designed as a cross-sectional study, and it was carried out on a sample of teenagers under the age of 18 who had been referred for orthodontic treatment to the Department of Orthodontics of the George Emil Palade University of Medicine, Pharmacy, Science, and Technology of Targu Mures. Subjects were selected from those who were registered between May 2024 and February 2025.

The exclusion criteria for the selection of our subjects were the following: systemic diseases or conditions, use of antibiotics within 3 months before the examinations, and periodontal lesions existing prior to bonding. In all cases, the same bracket system was used (Roth Omni, GAC). Molar tubes were bonded with 3M™ Transbond™ XT Light Cure Adhesive (C-composite) and, in fifteen cases, with a GC Fuji ORTHO™ LC Light-Cured Resin-Reinforced Glass Ionomer Orthodontic Adhesive (GIC—glass ionomer cementum). 

A microbiological sample was taken from the gingival sulcus of the maxillary first molars at the time of appliance placement (T1) and 6–8 weeks later (T2) at the first check-up. Patients were instructed and encouraged at the start of treatment to maintain proper personal oral hygiene, and their attention was drawn to the fact that, due to the bonded appliance, more attention should be paid to cleaning than usual.

The sampling was carried out as follows: The area was isolated with a cotton roll and air-dried. The supragingival biofilm was removed with a sterile curette, and the subgingival plaque sample was taken with sterile paper tips provided in the kit (micro-IDent Sampling Set, Hain Lifescience GmbH, Nehren, Germany). The paper tips were then placed in the transport tube and sent to the laboratory, where DNA extraction was performed (QIAamp^®^ DNA Mini Kit, Qiagen, Hilden, Germany). Molecular biology studies were performed at the Department of Microbiology, George Emil Palade University of Medicine, Pharmacy, Science, and Technology of Targu Mures.

The samples taken from the patients were tested using the micro-IDent^®^plus11 (Hain Lifescience GmbH, Germany) test, a PCR (polymerase chain reaction) method that allows for the extraction, amplification, and detection of bacterial DNA in the samples. This is an in vitro method that can detect the DNA of eleven periodontopathogenic bacteria: *Porphyromonas gingivalis* (P.g.), *Treponema denticola* (T.d.), *Tannerella forsythia* (T.f.), *Aggregatibacter actinomycetemcomitans* (A.a.), *Prevotella intermedia* (P.i.), *Fusobacterium nucleatum* (F.n.), *Campylobacter rectus* (C.r.), *Parvimonas micra* (P.m.), *Eubacterium nodatum* (E.n.), *Eikenella corrodens* (E.c.), and *Capnocytophaga* spp. (C.sp.).

The workflow can be divided into four steps: 1. DNA extraction (within seven days of sampling); 2. DNA amplification (amplification of double-stranded DNA extracted from samples, which is actually in vitro DNA synthesis); 3. DNA detection (detection of DNA, during which the amplified and denatured DNA binds to oligonucleotides that are fixed on nitrocellulose strips, based on the principle of complementarity); 4. interpretation (the test strips are interpreted using the evaluation sheet, and the obtained results are evaluated).

At the end of the process, the obtained results are interpreted and evaluated using the evaluation sheet. Each test strip has fifteen control zones:

- Conjugate Control (CC): This must be present, as it indicates the binding of the conjugate and the reaction of the substrate.
- Hybridization Control (HC): This must be present, as it indicates the completion of hybridization.
- Amplification Controls (AC1 and AC2): These must also be present, as they indicate the progress of the amplification process. The bands may be fainter in some cases, resulting from competition between amplicons. In the case of a negative result, when the band is missing, retesting is recommended.
- Specific Controls: These comprise eleven pieces, corresponding to the eleven periodontopathic bacteria that can be tested using the test. They must be read using the evaluation sheet that is included in the kit. The test result is negative if none of them appear but the control lines do, but if the control lines are also missing, we are faced with a false negative result, and the workflow must be repeated. In case of a positive result, the DNA of the bacteria that appear on the strip corresponds to the line.

**Figure 1:**
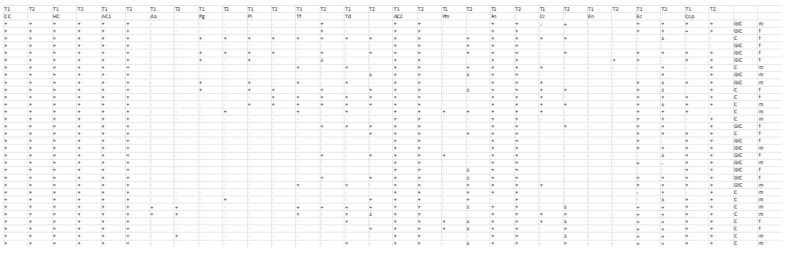
Identified periodontopathogens in the T1 and T2 samples.

The bacterial detection of the 11 species was assessed at two time points (T1 and T2) in two separate groups undergoing different bonding procedures: the GIC group and the C group. Data were recorded as binary outcomes, with “0” indicating absence and “1” indicating presence; ambiguous “±” values were recoded as “1” (present). For each bacterium, paired measurements from two time points (T1 and T2) were summarized in a 2 × 2 contingency table (Figure 1). McNemar’s test was then applied to these paired binary data to evaluate whether the detection rate changed significantly from T1 to T2 (Table 1). Both the chi-square approximation with continuity correction and the exact binomial test were employed, with statistical significance set at *p* < 0.05. This analysis was conducted separately for the GIC group and the C group.

We used Fisher’s Exact Test to compare the prevalence of each of the 11 bacteria at T1 and ultimately at T2 between the GIC group (15 patients) and the C group (16 patients). 

We applied the Bonferroni correction, setting the significance threshold at 0.05/11 ≈ 0.00455. The *p*-values below this threshold indicate a statistically significant difference in baseline prevalence between the two groups for that bacterium.

## 3. Results

The bacteria A.a., P.g., P.i., F.n., and E.n. showed no significant baseline differences between the GIC and C groups, with *p*-values of 1.000, indicating identical prevalence (either very high for Fn or 0% for En).

T.f., T.d., P.m., and C.r. had lower *p*-values (0.109, 0.066, 0.192, and 0.109, respectively), reflecting larger differences in prevalence (T.f.: 2/15 in GIC vs. 7/16 in C; T.d.: 3/15 in GIC vs. 9/16 in C; P.m.: 1/15 in GIC vs. 3/16 in C; C.r.: 2/15 in GIC vs. 7/16 in C), but none reached statistical significance.

E.c. had the lowest *p*-value (0.025), with a prevalence of 11/15 in GIC vs. 12/16 in C, but this was still not significant. C.sp., with a *p*-value of 0.72, also showed no significant difference. 

When comparing T1 and T2, the incidence of the 11 bacteria in the oral cavity of the 15 patients before and after the GIC bonding procedure revealed no statistically significant changes in bacterial presence, based on McNemar’s test (all exact *p*-values > 0.05). However, examining the trends in bacterial presence provided some insights into potential patterns and clinical considerations.

Across the 11 bacteria, there were 26 instances of a bacterium being gained (i.e., being absent at T1 but present at T2) and 14 instances of a bacterium being lost (i.e., being present at T1 but absent at T2), suggesting a net trend toward an increased bacterial presence in the oral cavity of the 15 patients. Specifically, there was an increase in the number of patients who tested positive for six out of the eleven bacteria: P.i. (seven gained, two lost), T.d. (six gained, one lost), T.f. (four gained, two lost), C.r. (three gained, two lost), E.n. (one gained, zero lost), and C.sp. (three gained, zero lost). Meanwhile, three bacteria showed a decrease: P.g. (two lost, zero gained), P.m. (two lost, zero gained), and E.c. (three lost, two gained). Finally, one bacterium showed no change: F.n. (all 15 patients had it at both T1 and T2). Despite these trends, the lack of statistical significance indicates that these changes could be due to chance and should be interpreted with caution. 

No significant difference was found in the prevalence of E.c. between the GIC (9/15) and C (16/16) groups at T2 after Bonferroni correction (*p* ≈ 0.0177). However, the *p*-value was the lowest at T2, and the difference in prevalence (60% in GIC vs. 100% in C) suggests that the C intervention may be less effective at controlling E.c., making E.c. a key bacterium to monitor. None of the differences at T2 were statistically significant after Bonferroni correction (threshold ≈ 0.00455), which is likely due to the small sample size (30 patients in total) and the low prevalence of some bacteria (e.g., E.n.: 1/31; A.a., P.g., and P.i.: 3/31 each at T2).

The Bonferroni correction is conservative, and the *p*-values for E.c. (0.0177), C.r. (0.135), and P.m. (0.142) were below the uncorrected threshold of 0.05, suggesting trends that may warrant further investigation in a larger study.

## 4. Discussion

The PCR method used in our research is a qualitative test that is suitable for examining the qualitative composition of the flora and its changes without providing any information on the number of bacteria; this means that it does not provide a quantitative result. Furthermore, the method does not distinguish between the DNA of living and dead bacteria but detects their presence equally.

Bacterial plaque is the main etiological factor in the development and progression of periodontal diseases. Orthodontic appliances are formed from a variety of biomaterials. The presence of increased amounts of biofilm increases the risk of gingivitis, and, as previously stated, this subsequently increases the risk of progression to periodontitis in susceptible individuals. The presence of orthodontic appliances can lead to increased colonization of certain bacterial species that are associated with periodontal diseases, such as P.g., A.a., and T.f. [5,6]. According to some researchers, the problem lies not only in the bacterial accumulation caused by the appliances but also in the composition of the subgingival microflora that is transformed into a more periodontogenic one, resulting in periodontitis over time. Meanwhile, it was confirmed that individual- and site-dependent microbiome variability exists in subgingival plaque [7]. Kado et al. reported a significant increase in periodontal pathogens (such as P.g. and P.i.) in supragingival plaque that was not transitory [8]. At the species level, P.i., C.r., F.n., and T.d. were elevated without significant differences, which might indicate that the subgingival plaque microbiome that is affected by fixed appliances could contribute to the transient mild gingival inflammation [9]. During the early stages, fixed appliance therapy influences the subgingival plaque microbial community, as reported by Guo et al.; these data are in accordance with the short-term results of Koopman et al. [10], who reported a significant microbial change in supragingival plaque after 6 weeks. The genus Veillonella was stable, while the genus Actinobacillus showed a temporary increase in the first month and then decreased with time. The relative abundances of *Prevotella intermedia* (P.i.) and *Campylobacter rectus* (C.r.) showed a temporary increase after 1 month of bonding and returned to the pretreatment level after 3 months of fixed appliance application. Slight increases in the relative abundances of *Fusobacterium nucleatum* (F.n.) and *Treponema denticola* (T.d.) were found after six months of bonding, while the changes in the relative abundances of the four periodontal pathogens were not statistically significant. The relative stability of the core microbiome and periodontal species (P.i.; C.r.; F.n.; T.d.) during the first three months of fixed appliance therapy might represent a relatively healthy periodontal status and suggest a risk of only transient periodontal inflammation in the early stages of fixed appliance therapy [9,10]. 

Our study suggests the following key observations: The C group consistently showed a higher prevalence of periodontal pathogens such as T.f., T.d., and C.r. at T1, suggesting a potential trend of a higher baseline periodontal risk in the C group, although this was not statistically significant. This could influence the interpretation of the T2 results, as the starting prevalence of these bacteria may affect how they change post-intervention. P.m.’s slight baseline difference is also noteworthy given its significant increase post-intervention in prior analyses, while E.c. and F.n., with high prevalences in both groups, are less likely to be distinguishing factors at baseline.

From a clinical perspective, the observed trend toward an increased presence of periodontal pathogens—particularly the bacteria in the red complex (T.d. and T.f., with 10 instances gained vs. 5 lost across the red complex), P.i., and C.sp.—might suggest that the procedure does not reduce these bacteria and could potentially create favorable conditions for their growth, such as biofilm formation on the restoration, or indicate limited antibacterial effects of glass ionomer cements against these species. The persistence of F.n. in all patients further supports the idea that the procedure has little impact on key periodontal pathogens. However, the slight decreases in P.g., P.m., and E.c. might hint at some minor inhibitory effect, possibly due to fluoride release, although this effect is not significant.

Given the lack of statistical significance, these trends do not confirm a definitive impact of the procedure on bacterial presence. However, they highlight the importance of clinical vigilance. Enhanced oral hygiene post-procedure (e.g., thorough brushing, flossing, and possibly antimicrobial mouthwashes such as chlorhexidine) can help manage any potential increase in periodontal pathogens. Patients with a history of periodontal disease should be monitored closely, as bacteria such as P.i. and T.d. showed notable (though not significant) increases. Clinicians might also consider polishing the glass ionomer restoration to reduce bacterial adhesion or exploring alternative materials with stronger antibacterial properties if pathogen reduction is a priority, especially for at-risk patients. Further research with a larger sample size is needed to determine whether these trends are meaningful and directly attributable to the procedure.

Analyzing some of the key patterns and trends regarding the higher prevalence of specific bacteria in the C Group at T2 (Table 1), the following observations were made: for A.a., P.g., P.i., T.d., P.m., C.r., and E.c., the C group showed higher or equal prevalences at T2 compared with the GIC group. 

This pattern suggests a potential protective effect of the GIC intervention against these bacteria, as the C group consistently showed greater increases or maintained higher prevalences. E.c., P.m., and C.r. had the lowest *p*-values (0.0177, 0.142, and 0.135, respectively), indicating the most notable differences, although none were significant after Bonferroni correction (threshold ≈ 0.00455).

In the GIC group at T2, T.f. and E.n. showed higher prevalences at T2 compared with the C group: T.f.: 2/15 to 7/15 (GIC) vs. 7/16 to 5/16 (C) and E.n.: 0/15 to 1/15 (GIC) vs. 0/16 at both T1 and T2 (C).

This reversal suggests that the GIC intervention may be less effective at controlling T.f. and E.n. For T.f., the increase in the GIC group (from 13.3% to 46.7%) and the decrease in the C group (from 43.8% to 31.3%) are particularly notable, although not significant (*p* ≈ 0.473). For E.n., the very low prevalence (1/31 at T2) limits the ability to detect any difference (*p* = 1.000).

F.n. and C.sp. showed no differences between the groups at T2, with very high prevalences in both groups at both time points: the prevalence of F.n. was 100% in both groups at T1 and T2 (*p* = 1.000), while that of C.sp. was 93.3% in the GIC group and 87.5% in the C group at both T1 and T2 (*p* = 1.000). These bacteria are likely core components of the oral microbiota in this cohort and unaffected by either intervention.

The following bacteria might be considered notable based on their *p*-values:

E.c. (*p* ≈ 0.0177): This bacterium achieved the lowest *p*-value at T2, with a prevalence of 60% in the GIC group vs. 100% in the C group, suggesting that C intervention is less effective at controlling Ec.

C.r. (*p* ≈ 0.135): This bacterium achieved the second-lowest *p*-value, with a prevalence of 20% in the GIC group vs. 50% in the C group, which is consistent with a higher baseline prevalence in the C group.

P.m. (*p* ≈ 0.142): This bacterium achieved the third-lowest *p*-value, with a dramatic increase in prevalence (4/31 at T1 to 19/31 at T2), particularly in the C group (75% vs. 46.7% in the GIC group), making Pm a key marker of intervention efficacy.

These three bacteria (E.c., C.r., and P.m.) show the most pronounced differences between groups, although none are significant after Bonferroni correction. 

When the effects of different types of orthodontic appliances (labial, lingual fixed appliances, and aligners) on the microbiomes were analyzed, most bacterial species showed moderate counts, except for T.d., which showed a higher count in all three types of appliances. F.n., P.g., and T.d. were present at higher percentages in the lingual appliance. F.n. and P.i. were present at higher percentages in the labial fixed appliance. C.r., T.f., and P.m. counts were moderately present in all three appliances, with the first two pathogens showing slightly higher counts in aligners. The associations between all the microorganisms were statistically insignificant, except for F.n., which showed a strong statistically significant association in all three types of appliances [11]. Additionally, there was an increase in gingival inflammation due to both the heightened bacterial load and the mechanical action exerted by the orthodontic appliances themselves [12]. The expression of “red-complex bacteria” in the dental plaque of patients undergoing orthodontic treatment was higher, irrespective of the presence or absence of gingival enlargement. Thorough oral hygiene maintenance is mandatory in patients undergoing fixed orthodontic treatment to prevent the risk of periodontal disease onset [13]. Studies on P.g. and A.a. have reported that one month after bracket removal, the contents of both bacteria were significantly decreased, and PCR detection of P.g. and A.a. in saliva revealed a certain level of these bacteria even after 6 months. After debonding, the contents of Pg and Aa decreased, and the content of Aa in the mouth was higher than that of P.g. [14]. The maximum levels of P.g., T.d., and T.f. were observed after debonding, while the smallest growth of these bacteria was observed eight weeks after debonding [15]. 

Analyzing both bonding procedures, some overall trends can be observed:

1. The GIC intervention appears to have a protective effect against A.a., P.g., P.i., T.d., P.m., C.r., and E.c., as these bacteria showed a higher prevalence in the C group at T2.
2. The GIC intervention may be less effective against T.f. and E.n., for which the GIC group showed higher prevalences at T2. F.n. and C.sp. are ubiquitous in this cohort, with no change in prevalence, making them non-distinguishing factors.
3. The small sample size (15 in the GIC group, 16 in the C group) and varying prevalence rates likely contributed to the lack of statistical significance, even for bacteria with notable differences (e.g., E.c., C.r., and P.m.).

The qualitative and quantitative changes in the microbial plaque of orthodontic patients occur as early as one week after the start of treatment, and they become more consistent three months after this, with stable colonization first by orange and then red species [2]. In fixed appliance therapy, the first changes occur already during the first weeks of treatment and within the first month, orange complex species predominate. After three months of bonding, the species in the orange complex increase and account for 40% of the total bacteria counts. The bacteria P.g., P.i., and T.f. in the red complex significantly increase after six months, as do Lactobacilli [16].

Based on our findings, some clinical implications can be identified:

1. The protective effect of GIC: The GIC intervention may offer some protection against A.a., P.g., P.i., T.d., P.m., C.r., and E.c., as these bacteria showed higher prevalence in the C group at T2. This could be due to GIC’s fluoride release or other antibacterial properties, which may inhibit the growth of these pathogens more effectively than the conventional cement (C).
2. The potential weakness of GIC against T.f. and E.n.: The higher prevalences of T.f. and E.n. in the GIC group at T2 suggest that GIC may be less effective at controlling these bacteria. T.f., a member of the “red complex” that is associated with severe periodontitis, is particularly concerning, as its increase in the GIC group (from 13.3% to 46.7%) could indicate a higher periodontal risk in that group post-intervention.

E.c., C.r., and P.m. can be considered key bacteria to monitor; hence, they are the most notable bacteria at T2 due to their low *p*-values and significant changes in prevalence. E.c.’s 100% prevalence in the C group, P.m.’s dramatic increase (especially in the C group), and C.r.’s persistent higher prevalence in the C group suggest that these bacteria may be useful markers for evaluating the efficacy of dental interventions in this context.

F.n. and C.sp. can be considered ubiquitous bacteria, with near-100% prevalences in both groups at both time points; they are likely core components of the oral microbiota in this cohort and are not influenced by the interventions. They do not provide insights into the differential effects of GIC vs. C treatment.

Even though our aim was to study the effects of bonding materials on the composition of subgingival microbiota, we are aware of the limitations of our study. One of them relates to the number of samples. Larger numbers of samples are needed for sound statistical significance, and increasing the spectrum of studied bacteria would also represent a better approach.

## 5. Conclusions

None of the 11 putative pathogens studied showed statistically significant differences due to the adhesive that was used for bonding.

GIC intervention appears to have a protective effect against A.a., P.g., P.i., T.d., P.m., C.r., and E.c., as these bacteria showed higher prevalences in the C group 6–8 weeks after bonding, and may be less effective against T.f. and E.n., for which the GIC group showed higher prevalence at T2. This finding may have an important effect on adhesive selection.

E.c., C.r., and P.m. can be considered key bacteria to monitor; hence, they are the most notable bacteria at T2 due to their low *p*-values and significant changes in prevalence. When a bonding material is selected, awareness regarding composite selection is essential. 

## Figures and Tables

**Table 1:** Prevalence of the 11 studied bacteria in the T1 and T2 samples.

Bacterium	T1			T2		
	*p*-Value (GIC vs. C)	Prevalence in GIC gr.	Prevalence in C gr.	*p*-Value (GIC vs. C)	Prevalence in GIC gr.	Prevalence in C gr.
A.a.	1	1/15	1/16	0.226	0/15	3/16
P.g.	1	5/15	4/16	0.192	1/15	3/16
P.i.	1	6/15	5/16	0.192	1/15	3/16
T.f.	0.109	2/15	7/16	0.473	7/15	5/16
T.d.	0.066	3/15	9/16	0.285	5/15	9/16
P.m.	0.192	1/15	3/16	0.142	7/15	12/16
F.n.	1	14/15	16/16	1	15/15	16/16
C.r.	0.109	2/15	7/16	0.135	3/15	8/16
E.n.	1	0/15	1/16	1	1/15	0/16
E.c.	0.025	11/15	12/16	0.0177	9/15	16/16
C.sp.	0.72	10/15	13/16	1	14/15	14/16

## Data Availability

The dataset analyzed during this article is available from the first author on request.
